# Supplementary material for: The Importance of Abnormal Platelet Count in Patients with Clostridioides difficile Infection
Source: J Clin Med. 2021 Jun 30;10(13):2957. doi: 10.3390/jcm10132957 (PMC8268691; doi:10.3390/jcm10132957)
Supplement: Supplementary file 1 [file jcm-10-02957-s001.zip › jcm-1227467-supplementary.pdf]

Supplementary Table S1. Univariate for Mortality

	DEAD 162	ALIVE 365	P VALUE, OR (CI)
Female	**77/285**	**85/242**	**0.04**
ADL Status	**121/294**	**41/233**	**0.000 3.27 (2.17-4.93)**
Housing LTCF	**44/108**	**118/419**	**0.012**
Dementia	**25/44**	**137/483**	**0.000**
IHD			0.28
CHF	**37/90**	**125/437**	**0.012**
COPD	**17/36**	**145/491**	**0.02**
CVA			0.07
CKD	**25/57**	**137/470**	**0.023**
DM			0.21
Malignancy			0.87
Haemato-oncology			0.82
SOT			0.31
Immunosuppression			0.91
Thrombocytopenia any	36/118	126/409	0.87 0.98 (0.63-1.53)
Stage 3	8/26	154/501	0.99
Stage 2	10/35	152/492	0.77
Stage 1	18/57	144/470	0.88
Thrombocytosis	**29/61**	**133/466**	**0.002 2.26 (1.32-3.89)**
Hypoalbuminemia	**102/218**	**59/304**	**0.000 3.65(2.47-5.38)**
Fidaxomicin			0.65
ICU	**8/12**	**154/515**	**0.006**
Colectomy			0.12
Age	**78+-12.4**	**67+-17.9**	**0.000**
Pulse	**93+-21**	**89+-18**	**0.006**
SAT	**94.4+-6.2**	**96.6+-3.5**	**0.000**
PLT	**281+-169**	**254+-146**	**0.03**
Creatinine	**1.53+1.18**	**1.1+-0.87**	**0.000**
BMI			0.116
Charlson's Score			0.261
Temperature			0.14
SBP			0.081
WBC			0.067
Hb			0.86
CRP			0.057
